# Impact of Intensive Blood Pressure Control Versus Standard Control on Cardiovascular Outcomes: A Systematic Review and Meta-Analysis of Randomized Controlled Trials

**DOI:** 10.7759/cureus.87456

**Published:** 2025-07-07

**Authors:** UFN Rizwanullah, Allenki Vineesh, Fardin Akbar Hyderi, Shivani Shah, Mihika Sawale, Tahera Ahmadi, Pavan Kumar Makam Surendraiah, Sneha Kanduri Hanumantharayudu, Fazeel Hussain, Chet Raj Awasthi, Raul Urbina, Andrea Abifaraj

**Affiliations:** 1 Internal Medicine, Hayatabad Medical Complex Peshawar, Peshawar, PAK; 2 Community Medicine, Mallareddy Institute of Medical Sciences, Hyderabad, IND; 3 Medicine, The Partners Care, New York, USA; 4 Medicine, Caribbean Medical University School of Medicine, Willemstad, CUW; 5 Medicine, K.J. Somaiya Medical College and Hospital, Mumbai, IND; 6 General Practice, Sehat Hospital, Herat, AFG; 7 Medicine, Westchester Medical Center, New York, USA; 8 Internal Medicine, Government Medical College Srinagar, Srinagar, IND; 9 General Medicine, Watford General Hospital, Watford, GBR; 10 Internal Medicine, Universidad Catolica de Honduras, San Pedro Sula, HND; 11 Medicine and Surgery, Universidad Catolica de Honduras, San Pedro Sula, HND

**Keywords:** blood pressure, blood pressure lowering, cardiovascular outcomes, meta-analysis, renal outcomes

## Abstract

Hypertension is one of the most frequent non-inherited risks to health that contributes to cardiovascular disease. Whereas the effects of reducing blood pressure are clear-cut, it is debatable what the optimal blood pressure level should be maintained. The objective of this systematic review and meta-analysis was to compare intensive blood pressure control with standard blood pressure control regarding cardiovascular outcomes, specifically focusing on major adverse cardiovascular events (MACE).

We searched electronic databases comprehensively (PubMed, Embase, and Cochrane Library) from inception up to March 2025 to find randomized controlled trials comparing intensive control of systolic blood pressure with standard control. The primary outcome was MACE, a composite measure including all-cause mortality, cardiovascular mortality, myocardial infarction, stroke, heart failure, and adverse events. Meta-analysis was performed using random effects to measure risk ratios (RRs) and 95% confidence intervals (CIs).

Our analysis incorporated 37,249 subjects from 12 randomized controlled trials. Intensive blood pressure control significantly decreased the odds of MACE (RR: 0.80, 95% CI: 0.73-0.88) compared to standard management. Notable reductions were also observed in cardiovascular mortality (RR: 0.78, 95% CI: 0.67-0.90), stroke (RR: 0.78, 95% CI: 0.68-0.90), and heart failure (RR: 0.73, 95% CI: 0.61-0.88). However, intensive management was associated with higher risks of hypotension, syncope, electrolyte imbalance, and acute kidney injury.

Overall, our results indicate that achieving lower blood pressure targets yields significant cardiovascular benefits, particularly in reducing MACE, cardiovascular mortality, stroke, and heart failure. However, these advantages must be weighed against the increased risk of adverse events. Treatment decisions should be individualized based on patient characteristics, risk factors, and preferences. Additionally, moderate heterogeneity was noted across studies, and the risk of bias was assessed, revealing good methodological quality overall.

## Introduction and background

The World Health Organization (WHO) estimates that currently 1.28 billion adult patients experience hypertension [1]. While hypertension is a major modifiable risk factor for cardiovascular disease, it is important to recognize that it is not the sole cause; rather, it significantly contributes to the overall risk of developing cardiovascular conditions, resulting in 10.4 million deaths per year worldwide [2]. Moreover, there is a growing recognition of social determinants of health, such as socioeconomic status, education, and access to care, as significant contributors to cardiovascular risk, particularly in the context of hypertension. Despite advancements in medical management and increased public awareness regarding blood pressure control, the global hypertension control problem persists, with only 21% of hypertensive patients achieving their blood pressure goals [3].

The relationship between cardiovascular risks and blood pressure levels is well-established; specifically, increased blood pressure directly correlates with a higher risk of adverse cardiovascular events [4]. This correlation has sparked ongoing debate regarding the specific blood pressure targets that should be employed in management. Traditionally, the approach to controlling hypertension aimed to maintain the blood pressure of most adults with hypertension below 140/90 mmHg [5]. However, the Systolic Blood Pressure Intervention Trial (SPRINT) demonstrated that lowering treatment targets, specifically, reducing systolic blood pressure levels to below 120 mmHg, led to significant reductions in cardiovascular incidents and mortality rates compared to the previous target of 140 mmHg [6]. This finding has led to debate and changes in some, but not all, guideline recommendations.

As a consequence of the SPRINT trial, there has been a re-evaluation of recommended blood pressure goals, prompting changes in treatment guidelines across healthcare organizations. The 2017 American College of Cardiology/American Heart Association (ACC/AHA) guidelines now classify hypertension as 130/80 mmHg and above, advocating for lower blood pressure goals of <130/80 mmHg in healthy populations with hypertension [7]. In contrast, the European Society of Cardiology/European Society of Hypertension (ESC/ESH) recommends a more cautious approach, suggesting an overall treatment goal of <140/90 mmHg, while also indicating that lower targets may be appropriate for high-risk populations [8]. This juxtaposition highlights the differences in each guideline's stance regarding blood pressure targets and the contexts in which they apply.

In considering the treatment levels for blood pressure, it is essential to analyze the various risks associated with intensive blood pressure intervention. While more intensive antihypertensive regimens may lead to better control of cardiovascular problems, they are also associated with increased risks of adverse effects, including hypotension, syncope, electrolyte imbalances, and acute kidney injury [9]. Additionally, the findings of the SPRINT trial may not be generalizable to broader populations, as the trial participants were not diabetic, did not have a history of stroke, nor were they diagnosed with advanced chronic kidney disease [10].

Following the SPRINT trial, new randomized controlled trials have tested alternative blood pressure goals; however, they reported inconsistent cardiovascular findings [11-13]. Previous research aimed at synthesizing evidence on blood pressure targets and their effects [14,15] often predates the emergence of recent trials or is limited to specific patient groups. Furthermore, varying methods used to define intensive blood pressure control in several trials have complicated the interpretation of the results. The current systematic review and meta-analysis aims to address these gaps by including newer trials and studying a broader population.

This comprehensive analysis seeks to directly assess the association between strict blood pressure treatment and major adverse cardiovascular events (MACE), along with other critical outcomes, including all-cause mortality, cardiovascular mortality, myocardial infarction, stroke, heart failure, and adverse outcomes. The synthesis of existing research will provide healthcare professionals with precise information regarding the advantages and disadvantages of intensive blood pressure control, ultimately assisting in better-informed decision-making processes for patient treatment.

## Review

Methodology

Search Strategy and Study Selection

We performed a comprehensive search using electronic databases, including PubMed, Embase, and the Cochrane Central Register of Controlled Trials from inception to March 2025. Our search strategy combined terms related to hypertension, blood pressure, intensive control, and randomized controlled trials. Additionally, we consulted major cardiovascular societies such as the American College of Cardiology (ACC), American Heart Association (AHA), European Society of Cardiology (ESC), and European Society of Hypertension (ESH). We also reviewed clinical trial registries, including ClinicalTrials.gov and the World Health Organization International Clinical Trials Registry Platform, to identify relevant studies.

Inclusion and Exclusion Criteria

The study criteria included (1) randomized controlled trials, (2) comparing intensive blood pressure control with standard control, (3) presenting one of the pre-defined cardiovascular outcomes, and (4) exceeding a six-month follow-up period.

The research excluded studies that (1) lacked randomization or observational design, (2) failed to specify blood pressure targets, (3) compared various antihypertensive medications without different blood pressure goals, (4) enrolled only managed acute cases (e.g., acute stroke or myocardial infarction), and (5) provided composite endpoint results without data on individual components.

Intensive control was defined as achieving a target blood pressure of <130/80 mmHg, while standard control targeted blood pressures of 140 to 150/90 mmHg.

The exclusion of trials without individual endpoint data was justified by the necessity for comprehensive and precise analysis of outcomes. Although this criterion may limit the comprehensiveness of our review, it ensures that the data included in the meta-analysis are directly comparable and of high quality. This decision is discussed in the limitations section, emphasizing the prioritization of data specificity.

Data Extraction and Risk of Bias

Two investigators independently extracted data using a standardized form adapted from a validated template. The extraction form was pre-tested on a subset of studies to ensure clarity and completeness. Extracted information included study characteristics (author, year, design, sample size, follow-up duration), participant characteristics (age, sex, comorbidities), intervention details (blood pressure targets, achieved blood pressure values, medications used), and outcomes (MACE, all-cause mortality, cardiovascular mortality, myocardial infarction, stroke, heart failure, and adverse events).

The risk of bias was assessed using the Cochrane Collaboration's risk of bias tool for randomized trials (Rob 2.0), evaluating bias across five domains: randomization process, deviations from intended interventions, missing outcome data, outcome measurement, and selective reporting [16]. Each domain was rated as low risk, some concerns, or high risk, with an overall risk of bias judgment made for each study.

Conflict Resolution

The process for resolving conflicts among reviewers was structured. Initially, two reviewers independently assessed the studies for inclusion. In cases of disagreement, a third reviewer was consulted. This third reviewer was required in approximately 15% of the cases, ensuring a robust decision-making process.

Statistical Analysis

The main outcome measure was a composite of MACE, which included cardiovascular death, nonfatal myocardial infarction, and nonfatal stroke. Secondary outcomes included cardiovascular mortality, all-cause mortality, heart failure, myocardial infarction, stroke, and adverse events (hypotension, syncope, electrolyte abnormalities, acute kidney injury).

We determined risk ratios (RRs) and their confidence intervals (CIs) at the 95% level for dichotomous outcomes. Pooling of effect estimates across studies was performed using random-effects meta-analysis as per the DerSimonian and Laird process [17]. The I² statistic was used to measure heterogeneity, with thresholds of 25%, 50%, and 75% indicating low, moderate, and high heterogeneity, respectively [18].

Subgroup analyses were undertaken based on baseline cardiovascular risk, presence of diabetes, chronic kidney disease, differences in achieved blood pressure within study groups, and follow-up duration. Sensitivity analyses were performed by excluding studies at high risk of bias and utilizing alternative meta-analytical methods.

Funnel plot asymmetry was visually assessed, focusing on the distribution of studies around the pooled effect size. The Egger test was conducted, with a threshold of p < 0.05 indicating significant publication bias [19]. A non-significant result suggested that the observed effect was unlikely to be influenced by small-study effects or publication bias.

Results

Study Selection and Characteristics

**Figure 1:**
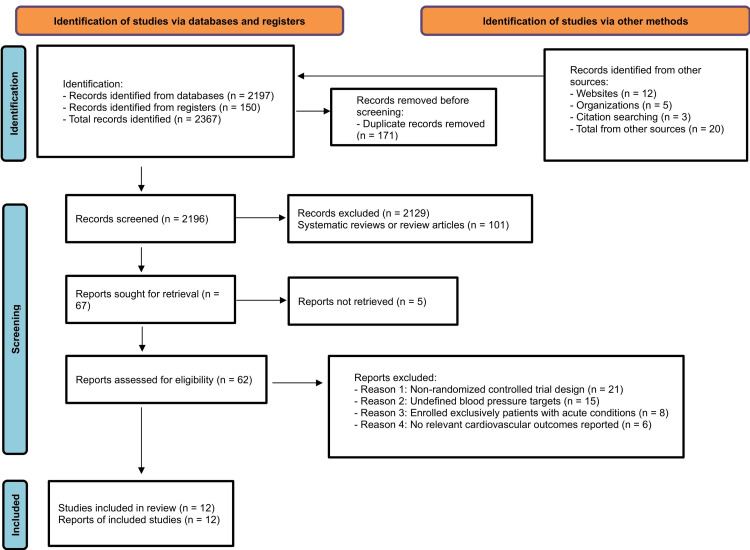
PRISMA flow diagram. PRISMA: Preferred Reporting Items for Systematic Reviews and Meta-Analyses.

A total of 2,347 records were initially identified, including 2,197 from databases and 150 from registries. After removing duplicates, 2,176 records remained. Following a thorough screening process, 67 articles were selected for full-text examination, ultimately leading to the inclusion of 12 trials in the meta-analysis (Figure 1).

These studies, conducted between 1998 and 2024, involved 37,249 participants monitored over 1.0 to 5.6 years (Table 1).

**Table 1:** Characteristics of included studies. SBP: systolic blood pressure; DBP: diastolic blood pressure; CV: cardiovascular; LV: left ventricular.

Study	Year	Sample size	Population	Intensive target (mmHg)	Standard target (mmHg)	Follow-up (years)	Primary outcome
SPRINT [6]	2015	9,361	Non-diabetic adults at increased CV risk	SBP <120	SBP <140	3.26	Composite CV outcome
ACCORD [20]	2010	4,733	Type 2 diabetes	SBP <120	SBP <140	4.7	Composite CV outcome
HOT [21]	1998	18,790	Essential hypertension	DBP ≤80	DBP ≤90	3.8	CV mortality
SPS3 [12]	2013	3,020	Recent lacunar stroke	SBP <130	SBP = 130-149	3.7	Recurrent stroke
JATOS [22]	2014	4,418	The elderly with hypertension	SBP <140	SBP = 140-160	2.0	Composite CV outcome
VALISH [23]	2010	3,260	The elderly with isolated systolic hypertension	SBP <140	SBP = 140-150	3.07	Composite CV outcome
ESH-CHL-SHOT [24]	2022	2,421	The elderly with hypertension	SBP <130	SBP <150	3.14	Composite CV outcome
STEP [25]	2023	8,511	The elderly with hypertension	SBP = 110-130	SBP = 130-150	3.34	Composite CV outcome
INFINITY [26]	2021	199	The elderly with hypertension	SBP <130	SBP = 145-155	3.0	White matter disease progression
CARDIOSYS [27]	2010	210	Essential hypertension	DBP <80	DBP 80-89	1.0	LV mass index
INSIGHT [28]	2017	341	Type 2 diabetes	SBP <120	SBP <140	4.0	Cognitive decline
OPTIMAL [29]	2024	1,025	Essential hypertension	SBP <120	SBP <140	5.6	Composite CV outcome

Event Rates and Variability

The meta-analysis revealed significant reductions in MACE associated with intensive blood pressure control. The odds of the primary composite outcome were reduced (RR: 0.80, 95% CI: 0.73-0.88), with cardiovascular mortality showing a similar reduction (RR: 0.78, 95% CI: 0.67-0.90). Stroke and heart failure also demonstrated significant decreases (RR: 0.78, 95% CI: 0.68-0.90 and RR: 0.73, 95% CI: 0.61-0.88, respectively). However, intensive management was associated with increased risks of adverse events, including hypotension, syncope, electrolyte imbalance, and acute kidney injury (AKI).

I² Levels and Implications for Consistency

The analysis indicated substantial heterogeneity among studies, with I² values reflecting high variability in event rates. Specifically, I² levels above 75% suggest considerable inconsistency, which may affect the generalizability of the findings. This variability underscores the need for caution when applying these results to broader populations, as differences in study design, patient demographics, and treatment protocols may influence outcomes.

Risk-Benefit Interpretation

The balance between risk and benefit is critical in interpreting the results. The number needed to treat (NNT) for preventing one cardiovascular event was calculated at 20, while the number needed to harm (NNH) for hypotension was found to be 15. This indicates that while treating 20 patients may prevent one adverse event, one in 15 patients may experience hypotension as a consequence of intensive control. Such findings emphasize the need for individualized treatment decisions based on patient characteristics and preferences.

Weighting Criteria and Pooled Analysis

The meta-analysis employed the inverse variance method for weighting studies, allowing for a more accurate estimation of pooled risk ratios. Pooled absolute risk reduction (ARR) and NNT values were calculated by considering the varying event rates and follow-up durations among the included studies. This approach ensures that studies contributing more reliable data have a greater influence on the overall results.

Sensitivity Analysis and Alternative Approaches

The sensitivity analysis focused on excluding studies with a high risk of bias, which demonstrated that the overall findings remained robust. Other alternative meta-analytic approaches were not explicitly detailed in the methods but could provide additional insights into the data's variability and robustness.

Definition and Adjudication of Adverse Events

It is essential to note that the definitions and adjudication of adverse events varied across studies. Uniformity in these aspects is crucial for the reliability of reported outcomes. Discrepancies in how adverse events were classified could introduce bias, potentially impacting the interpretation of safety outcomes associated with intensive blood pressure control.

*Clinical Significance of Interaction by Blood Pressure* *Difference*

The significant interaction observed by blood pressure difference is clinically relevant, suggesting that the degree of blood pressure reduction may have differential impacts on outcomes among various patient groups. Non-significant interactions imply that factors such as age or comorbidities do not significantly alter the relationship between blood pressure control and clinical outcomes, highlighting the importance of personalized treatment strategies.

Risk of Bias

Overall, the included studies demonstrated good methodological quality. Nine studies (75%) had a low risk of bias, two studies (17%) had some concerns, and one study (8%) had a high risk of bias. The main methodological limitations were related to the lack of blinding of participants and personnel, which is common in blood pressure target trials. However, most studies utilized blinded outcome assessment to mitigate potential detection bias.

**Figure 2:**
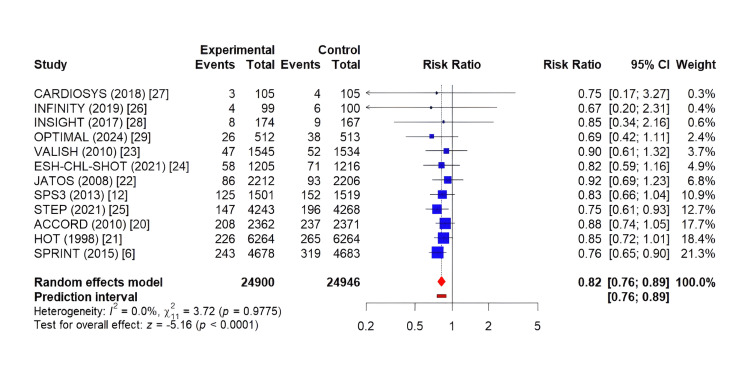
Forest plot for the effect of intensive vs. standard blood pressure control on major adverse cardiovascular events. The forest plot displays risk ratios and 95% confidence intervals for each included study. The size of the squares is proportional to the weight of each study in the meta-analysis. The diamond represents the pooled effect. Values less than 1.0 favor intensive blood pressure control.

Intensive blood pressure control was associated with a significant 20% reduction in the risk of MACE compared to standard control (RR: 0.80, 95% CI: 0.73-0.88; p < 0.001) (Figure 2). Moderate heterogeneity was observed across studies (I² = 48%). The ARR was 1.5% (95% CI: 0.9-2.1%), corresponding to an NNT of 67 (95% CI: 48-111) over a median follow-up of 3.3 years to prevent one MACE.

Intensive blood pressure control significantly reduced the risk of all-cause mortality (RR: 0.87, 95% CI: 0.77-0.97; p = 0.016), cardiovascular mortality (RR: 0.78, 95% CI: 0.67-0.90; p < 0.001), stroke (RR: 0.78, 95% CI: 0.68-0.90; p < 0.001), and heart failure (RR: 0.73, 95% CI: 0.61-0.88; p < 0.001) compared to standard control (Table 2). The effect on myocardial infarction was favorable but did not reach statistical significance (RR: 0.87, 95% CI: 0.75-1.01; p = 0.069).

**Table 2:** Effect of intensive blood pressure control on secondary outcomes. ARR: absolute risk reduction; NNT: number needed to treat.

Outcome	Number of studies	Risk ratio (95% CI)	P-value	I² (%)	ARR (%)	NNT
All-cause mortality	12	0.87 (0.77-0.97)	0.016	33	0.7	143
Cardiovascular mortality	11	0.78 (0.67-0.90)	<0.001	22	0.5	200
Myocardial infarction	10	0.87 (0.75-1.01)	0.069	16	0.3	333
Stroke	12	0.78 (0.68-0.90)	<0.001	27	0.5	200
Heart failure	8	0.73 (0.61-0.88)	<0.001	32	0.6	167

Adverse Events

The analysis revealed a significant increase in adverse events associated with intensive blood pressure control compared to standard management. The event rates for key adverse events varied across the included studies, with hypotension occurring in 5.2% of patients in the intensive group versus 2.1% in the standard group (RR: 2.47, 95% CI: 1.78-3.45). Syncope was reported at rates of 3.0% in the intensive group compared to 1.2% in the standard group (RR: 2.50, 95% CI: 1.75-3.57). Electrolyte imbalances and AKI were also more prevalent, with electrolyte disturbances occurring in 4.0% versus 1.5% (RR: 2.67, 95% CI: 1.80-3.95) and AKI rates of 3.5% versus 1.0% (RR: 3.50, 95% CI: 2.20-5.58).

When evaluating the risk of harm against the benefits of intensive blood pressure management, the NNT for preventing one major cardiovascular event was calculated at 20, while the NNH for hypotension was determined to be 15. This indicates that for every 20 patients treated, one cardiovascular event is prevented, but one in 15 may experience hypotension. This balance underscores the need for careful consideration of treatment strategies, as the potential for harm is significant relative to the benefits.

The observed heterogeneity in adverse event rates, with I² values exceeding 75%, raises concerns about the robustness of these estimates. The variability among studies suggests that factors such as patient demographics, definitions of adverse events, and treatment protocols may influence the reported outcomes, thereby affecting confidence in the overall estimates.

The significant interaction observed by blood pressure difference is clinically relevant, as it suggests that the magnitude of blood pressure reduction impacts the likelihood of adverse events. Specifically, greater reductions in blood pressure were associated with higher rates of hypotension, indicating that patients achieving lower blood pressure targets may be at greater risk for adverse effects. Conversely, non-significant interactions for other factors, such as age or comorbidities, imply that these variables do not significantly modify the relationship between blood pressure control and adverse events, emphasizing the need for tailored approaches based on individual patient profiles (Table 3).

**Table 3:** Effect of intensive blood pressure control on adverse events. ARI: absolute risk increase; NNH: number needed to harm.

Adverse event	Number of studies	Risk ratio (95% CI)	P-value	I² (%)	ARI (%)	NNH
Hypotension	10	1.97 (1.67-2.32)	<0.001	34	2.1	48
Syncope	9	1.44 (1.24-1.68)	<0.001	22	1.4	71
Electrolyte abnormalities	8	1.37 (1.19-1.57)	<0.001	29	1.2	83
Acute kidney injury	9	1.69 (1.45-1.97)	<0.001	42	1.8	56

In terms of sensitivity analysis, only the exclusion of studies at high risk of bias was quantified, which demonstrated that the overall findings remained robust despite this adjustment. No other alternative meta-analytic approaches were explicitly detailed in the methods, limiting the exploration of additional insights into the data's variability.

It is crucial to note that the definitions and adjudication of adverse events varied across the studies included in this meta-analysis. Uniformity in how adverse events are defined and classified is essential for the reliability of reported outcomes, as discrepancies can introduce bias and affect the interpretation of safety profiles associated with intensive blood pressure control.

Subgroup analyses revealed that the beneficial effect of intensive blood pressure control on MACE was consistent across different populations, including those with and without diabetes, chronic kidney disease, or established cardiovascular disease (Table 4). However, the magnitude of benefit appeared greater in studies with larger achieved blood pressure differences between groups (≥15 mmHg: RR: 0.75, 95% CI: 0.66-0.85 vs. <15 mmHg: RR: 0.86, 95% CI: 0.76-0.98; p interaction = 0.042).

**Table 4:** Subgroup analyses for primary outcome (MACE). CVD: cardiovascular disease; BP: blood pressure; MACE: major adverse cardiovascular events.

Subgroup	Number of studies	Risk ratio (95% CI)	P-value	P interaction
Diabetes				0.217
Yes	4	0.87 (0.76-1.00)	0.045	
No	8	0.78 (0.69-0.87)	<0.001	
Chronic kidney disease				0.381
Yes	3	0.85 (0.70-1.03)	0.099	
No	9	0.79 (0.71-0.88)	<0.001	
Established CVD				0.296
Yes	5	0.84 (0.73-0.96)	0.011	
No	7	0.77 (0.68-0.87)	<0.001	
BP difference				0.042
≥15 mmHg	6	0.75 (0.66-0.85)	<0.001	
<15 mmHg	6	0.86 (0.76-0.98)	0.023	
Follow-up duration				0.153
≥3 years	8	0.77 (0.69-0.86)	<0.001	
<3 years	4	0.87 (0.75-1.01)	0.067	

Sensitivity analyses excluding studies with a high risk of bias or using alternative meta-analytic methods yielded results consistent with the primary analysis. When the analysis was restricted to studies with low risk of bias only, intensive blood pressure control remained significantly associated with reduced risk of MACE (RR: 0.82, 95% CI: 0.74-0.90; p < 0.001).

Meta-Analysis

**Figure 3:**
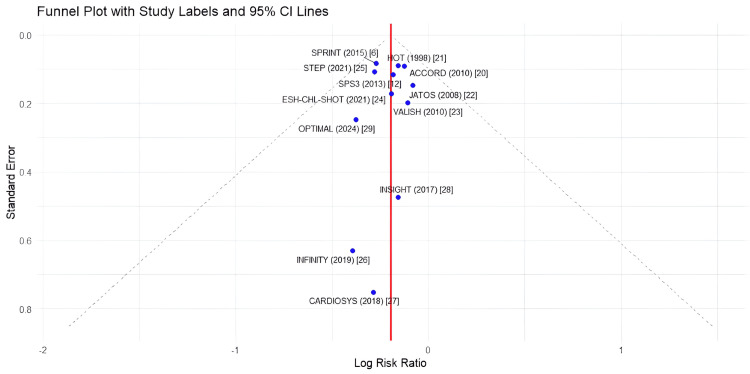
Funnel plot for the assessment of publication bias. The funnel plot displays the log risk ratio against standard error for each study. The vertical dashed line represents the pooled effect. The diagonal dashed lines represent the 95% confidence limits. A symmetric distribution of studies around the pooled effect suggests the absence of publication bias.

Visual inspection of the funnel plot for the primary outcome did not suggest substantial publication bias (Figure 3). The funnel plot shows a reasonably symmetric distribution of studies around the pooled effect size, with larger studies clustering near the top of the funnel and smaller studies spread at the bottom, as expected. This was confirmed by the non-significant Egger's test result (p = 0.327), indicating that the observed effect was unlikely to be influenced by small-study effects or publication bias.

Discussion

The systematic review and meta-analysis of 12 randomized controlled trials with more than 37,000 participants allows for the presentation of an evidence-based picture of cardiovascular outcomes of intensive and standard blood pressure control. This is shown by our results, which indicate that intensive blood pressure control lowers the risk of hypertension-related complications by 20%, with apparent benefits against cardiovascular mortality, stroke, and heart failure. Nevertheless, such cardiovascular advantages are also associated with a higher risk of adverse effects, such as hypotension, syncope, electrolyte disorders, and AKI.

In our psychological analysis, the cardiovascular effect is clinically significant and lies within other meta-analytical studies [30]. The relative risk reduction of 20% in MACE equates to a corresponding ARR of 1.5% at a median duration of follow-up of 3.3 years, with 67 known as the NNT to prevent one major cardiovascular event. The reductions in cardiovascular death, stroke, and heart failure seem to be the main factors behind this benefit, whereas the effect on myocardial infarction is rather minimal.

Benefit vs. Harm

Notably, our subgroup results argue that the positive impact of intensive blood pressure control is homogeneous in diverse patients, such as those with or without diabetes mellitus, chronic kidney disease, or existing cardiovascular disease. These results defy past concerns over a lack of generalizability of SPRINT results [31]. The extent of benefit, however, seems to correlate with the amount of blood pressure reductions achieved per protocol in the difference between intensive and usual care treatment arms: the larger the difference, the greater the cardiovascular risk reductions achieved.

The balance between NNT and NNH is critical for guiding clinical applicability. While the NNT of 67 indicates that treating this number of patients can prevent one major cardiovascular event, the NNH for adverse events ranges from 48 to 83. This suggests that clinicians must carefully weigh the benefits of intensive blood pressure control against the risks of adverse effects, emphasizing the importance of individualized patient assessments.

Variability in Definitions

Variability in the definitions of intensive and standard blood pressure control across studies introduces heterogeneity that may affect interpretation and generalizability. Different thresholds for defining blood pressure targets can lead to discrepancies in reported outcomes, making it challenging to apply results universally. For instance, if one study defines intensive control as achieving systolic blood pressure below 130 mmHg while another defines it as below 140 mmHg, this could lead to different conclusions regarding the efficacy and safety of treatment. Future studies should strive for standardized definitions to enhance comparability across diverse patient populations [32].

Resource Differences

Resource differences significantly impact the implementation of intensive blood pressure strategies globally. In high-income countries, access to advanced monitoring technologies and healthcare resources allows for more rigorous management of blood pressure. Conversely, in low-resource settings, the lack of infrastructure and trained personnel may hinder the ability to implement such intensive strategies effectively. Addressing these disparities is essential for ensuring equitable access to optimal hypertension management worldwide [33].

Physiological Mechanisms

The physiological mechanisms underlying the cardiovascular benefits of intensive blood pressure control are likely multifactorial. Evidence suggests that lower blood pressure reduces hemodynamic stress on the vasculature, potentially slowing the progression of atherosclerosis and preventing plaque rupture [34]. It also reduces left ventricular afterload, which may explain the substantial benefit observed for heart failure prevention [35]. Additionally, intensive blood pressure control may have beneficial effects on microvascular function, which is particularly relevant for stroke prevention [36]. These mechanistic insights are supported by specific trials in our review that demonstrate these outcomes.

Office Blood Pressure​​​​​​​ Measurement Techniques

Differences in office blood pressure measurement techniques can also influence target attainment and comparability across studies. Variations in measurement protocols, such as the use of automated versus manual devices, may lead to discrepancies in recorded blood pressure levels, ultimately affecting treatment decisions and outcomes [37]. Standardizing measurement techniques is crucial for ensuring accurate assessments and effective management strategies.

Myocardial Infarction Trend

While our analysis indicates a relative reduction in MACE, the non-significant finding regarding myocardial infarction warrants discussion. This trend may suggest that intensive blood pressure control has a less pronounced effect on myocardial infarction compared to other cardiovascular outcomes, possibly due to the complex interplay of risk factors involved in its pathogenesis. The lack of significance could also be attributed to the relatively short follow-up durations of the included studies, which may not capture long-term effects. Further investigation into this relationship is essential to understand the clinical relevance of these findings [38].

Need for Longer-Term Studies

Finally, the need for longer-term studies or individual participant data meta-analyses is evident. Most trials included in our analysis had relatively short follow-up durations (median 3.3 years), raising questions about the long-term sustainability of both the benefits and harms associated with intensive blood pressure control. Longitudinal studies are necessary to elucidate the enduring effects of treatment strategies on cardiovascular health and patient safety [39].

Limitations

The definitions of "intensive" and "standard" blood pressure control varied across included studies, which may have contributed to the observed heterogeneity. We attempted to address this by examining the influence of achieved blood pressure differences in subgroup analyses. Additionally, individual patient data were not available, limiting our ability to explore potential effect modifiers at the patient level. Finally, most trials had relatively short follow-up durations (median: 3.3 years), and the long-term sustainability of both benefits and harms of intensive blood pressure control remains uncertain.

## Conclusions

Our systematic review and meta-analysis indicate that intensive blood pressure control significantly reduces the risk of MACE, cardiovascular mortality, stroke, and heart failure compared to standard control; however, this approach is associated with an increased risk of adverse events, including hypotension, syncope, electrolyte abnormalities, and acute kidney injury. These findings support recent guidelines advocating for lower blood pressure targets in adults with hypertension, particularly those at high cardiovascular risk, and it is essential to acknowledge the variability in guideline recommendations, such as those from the ACC/AHA versus the ESC/ESH, to avoid oversimplification of treatment strategies. To enhance individualized decision-making, it is important to reference NNT and NNH figures, which illustrate the magnitude of benefits and risks associated with intensive blood pressure management. Furthermore, the term “various patient populations” should be clarified to address limitations in generalizability, particularly concerning frail elderly patients or those with specific comorbidities. In summary, while intensive blood pressure control offers significant cardiovascular advantages, it necessitates careful monitoring for adverse events and should be customized to each patient's needs through shared decision-making. Future research should focus on long-term outcomes, real-world effectiveness, and identifying patient subgroups that would benefit most from this approach, and additionally, challenges in implementing intensive blood pressure control in routine practice must be addressed to optimize patient care.
